# Personalized Intelligent Chatbot Based on AI-Generated Content Assists Memoir Writing for Older Adults With Cognitive Impairment: Mixed Methods Study

**DOI:** 10.2196/83428

**Published:** 2026-08-25

**Authors:** Yibo Meng, Yuan Que, Zhe Yan, Bingyi Liu, Zixin Wang, Mandi Yang, Huidi Lu

**Affiliations:** 1 Tsinghua University, Beijing, China; 2 Southwest Jiaotong University, Chongqing, China; 3 The Chinese University of Hong Kong, Shenzhen, Shenzhen, China; 4 University of Michigan, Ann Arbor, Ann Arbor, MI, United States; 5 University of Pennsylvania, Philadelphia, PA, United States; 6 Nankai University, Tianjin, China; 7 Said Business School, University of Oxford, Park End Street, Oxford, England, OX1 1HP, United Kingdom, +44 1865 288800

**Keywords:** AI-generated content, older adults with dementia, memoir writing, multimethod design, conversational robot, cognitive impairment

## Abstract

**Background:**

Older adults with cognitive impairment often face significant challenges in memoir writing, including memory fragmentation, emotional loneliness, and speech and language disorders. Although AI-generated content (AIGC) technologies such as GPT-3.5 show potential in creative tasks, they often lack the personalization and adaptability required for users with dementia. Generic AIGC tools often fail to address the heterogeneous cognitive and emotional needs of this population.

**Objective:**

This study aimed to design and evaluate, as a proof-of-concept, a personalized AIGC-powered chatbot to assist older adults with cognitive impairment in memoir writing and emotional support.

**Methods:**

We developed a multimethod collaborative design framework integrating Kansei Engineering, Quality Function Deployment, Axiomatic Design, and the Technique for Order of Preference by Similarity to Ideal Solution decision model. The system dynamically adapts interaction strategies based on users’ Mini-Mental State Examination (MMSE) scores, using a clinical threshold of 20 to distinguish mild (20-26) from moderate-to-severe (<20) impairment. In a single-session, nonrandomized, matched-pair evaluation using minimization-based allocation with an active control condition, performance was assessed via usability testing (System Usability Scale), affect assessment (Positive and Negative Affect Schedule), and blinded psychiatrist-rated memoir quality among 20 participants (10 per arm).

**Results:**

The experimental group showed significantly greater improvement than the active control group in positive affect, negative affect, and psychiatrist-rated memoir quality (all *Ps*≤.03 by matched-pair analysis; all comparisons remained significant after Benjamini-Hochberg correction), with moderate-to-large effect sizes (Cohen *d*=0.85‐1.03 for affect outcomes; rank-biserial *r*=1.00 for memoir quality). Within the sample (MMSE range 11‐23), participants in the lower MMSE tier (<20; n=12) appeared to benefit more from AI-driven narrative generation, while those in the higher tier (20-23; n=8) responded better to keyword-based prompting; these subgroup observations are descriptive, given the small cell sizes. The mean System Usability Scale total score of 92.2 (SD 5.1) exceeded the acceptability threshold and fell in the excellent range, though single-session exposure and potential acquiescence bias warrant cautious interpretation.

**Conclusions:**

These findings provide preliminary, hypothesis-generating evidence that a personalized AIGC-based chatbot may support short-term affective and narrative outcomes among older adults with cognitive impairment. The adaptive, multimodal design shows promise for human-AI collaboration in memoir writing and older adult care contexts, but larger, adequately powered randomized controlled trials with verified active control fidelity, multisession follow-up, and content source–differentiated outcome scoring are needed before clinical conclusions can be drawn.

## Introduction

Dementia affects over 50 million individuals globally, impairing memory, cognition, language, and emotional processing, and this number is projected to grow significantly with the aging population [1]. Among nonpharmacological interventions, memoir writing has been shown to support cognitive function and emotional healing by preserving personal narratives and enhancing identity [2,3]. AI-driven approaches to structuring fragmented life narratives have shown early technical promise in organizing and preserving personal stories for therapeutic use [4]. However, traditional self-narration methods impose high cognitive demands, particularly for individuals with language difficulties and fragmented memory [5]. Emerging AI-generated content (AIGC) technologies such as GPT-3.5 offer new opportunities for content generation with minimal user input [5,6]; yet, most current systems are not designed to adapt to the cognitive and emotional needs of people with dementia.

By 2050, the global prevalence of dementia is projected to exceed 152 million [1]. Memoir writing, as a fundamental nonpharmacological intervention, has been increasingly validated for its dual benefits in cognitive preservation and emotional healing [2,3]. Nonetheless, the cognitive impediments (eg, memory fragmentation) and emotional requirements (eg, loneliness) of this demographic are predominantly unaddressed by current technologies [5,7]. Generic AIGC tools do not possess the necessary adaptability for dementia care [7]. This research gap highlights the urgent need for personalized, cognition-adaptive AIGC solutions. In addition to cognitive support, memoir writing functions as a means of preserving narrative identity and dignity [8,9]. Bitenc [9] emphasized that coherent life narratives play a crucial role in preserving selfhood in individuals with dementia, reinforcing the emphasis on both cognitive and emotional support in the chatbot design.

Recent studies in human-computer interaction (HCI) emphasize the role of asymmetric collaboration [10,11], where AI systems adapt dynamically to users with varying cognitive abilities. Assistive technologies can act as “ability balancers,” helping patients with dementia engage meaningfully despite limitations [12-14]. Yet, most existing tools focus on functional or informational support and do not address the emotional and narrative dimensions central to memoir writing. AIGC tools like Replika have been explored for emotional companionship and mental health applications [15,16], but they often lack medical adaptability and rigorous ethical safeguards required in dementia care [17]. Existing dementia-focused chatbots have primarily targeted family caregivers rather than patients themselves [18], leaving a gap in direct patient-facing conversational support. Traditional dementia-assistive technologies emphasize personalization and usability [7,19], but seldom provide real-time narrative generation or emotion-sensitive feedback.

To address these limitations, we propose a personalized AIGC-powered chatbot that dynamically adjusts its interaction based on user cognitive states measured by Mini-Mental State Examination (MMSE) scores [20] and responds to emotional needs using Kansei Engineering (KE) principles [21]. The system integrates methods from mechanical engineering, packaging design, and HCI, aiming to support memoir writing, improve emotional well-being, and enable adaptive, human-centered AI experiences for older adults with cognitive impairment. This interdisciplinary design adheres to contemporary evidence on nonpharmacological interventions in dementia, which have demonstrated efficacy in improving emotional well-being [22,23], and addresses the demand for emotion-centric and cognition-adaptive assistive technologies in HCI and gerontechnology research [24,25].

This paper makes 3 contributions. First, we present a cognition-adaptive AIGC chatbot designed specifically to support memoir writing among older adults with cognitive impairment. The system combines MMSE-based interaction control with a structured life-event memory system and caregiver-verified memory anchors. Second, we describe a reproducible multimethod design process integrating KE, Quality Function Deployment (QFD), Axiomatic Design (AD), and Technique for Order of Preference by Similarity to Ideal Solution (TOPSIS) to translate affective and cognitive needs into system specifications. Third, we report a preliminary matched-pair evaluation involving 20 participants, using a minimization-based allocation procedure to examine short-term changes in affect, psychiatrist-rated memoir quality, and perceived usability. The study is intended as a proof-of-concept evaluation of the system’s feasibility and potential rather than evidence of clinical efficacy. Additional design details are provided in Multimedia Appendix 1.

## Methods

### Research Objectives

This study aimed to design and evaluate a personalized AIGC-based chatbot to support older individuals with cognitive impairment in memoir writing and emotional support. The system dynamically adapts its interaction mode based on users’ MMSE scores, offering keyword prompts for those with mild impairment and active narrative generation for those with severe cognitive decline. The chatbot identifies and responds to emotional needs such as loneliness and the desire for achievement through KE principles. Following system design, a user evaluation study assessed effectiveness.

### System Design

#### Design Framework

To develop a personalized AIGC system supporting memoir writing for older individuals with dementia, we adopted a multistage collaborative design framework integrating KE, QFD, AD, and the TOPSIS decision model.

In the first stage, KE was applied to identify users’ emotional and psychological needs, drawing on the dementia-care literature on memory loss, caregiver burden, and identity disruption to derive core affective themes such as “frustration from forgetting” and “the desire for recognition.” In the second stage, QFD mapped these emotional needs to quantifiable technical specifications, such as ensuring a natural language processing response delay of less than 2 seconds and incorporating a comforting tone in audio feedback. In the third stage, AD decomposed the system into 3 functionally independent but complementary modules: memory triggering, narrative generation, and emotional feedback. Finally, the TOPSIS method evaluated and selected among 3 intervention strategies (low, medium, and high AI proactivity), with emotional needs weighted more heavily than technical complexity to prioritize emotionally resonant interactions.

This multimethod framework is supported by existing literature. Li and Li [26] demonstrated that combining KE, QFD, and AD can improve user satisfaction in medical product design for older adults, providing empirical support for this multimethod approach. The emotional needs mapping in the QFD phase aligns with the finding of Raghunath et al [24] that translating user needs into structured design specifications can meaningfully reduce cognitive demands on users. The emphasis on emotional needs in the TOPSIS model is further supported by ethical research underscoring emotional well-being as a fundamental outcome in dementia care [23].

#### System Implementation

The system uses GPT-3.5 as its core AIGC engine, integrated within a companion device intended for home use. The device’s main controller processor coordinates a Wi-Fi module for wireless connectivity; ultrasonic sensors and an indicating light for environmental sensing and status feedback; a speech recognition chip linked to a microphone for voice input; a speech decoding and playback module linked to a horn for audio output and to a USB flash drive, TF card, and flash memory for local audio storage; and a TC117 driving chip powered jointly by a battery and an SY8101B power module. Interaction is controlled through prompt engineering: high-level prompt templates define the system’s role, task objectives, and interaction norms, with placeholders reserved for cognitive state variables. A typical template reads: “You are a memoir construction assistant for older adults. Based on the user’s cognitive level (MMSE score: [X]), guide them to elaborate on life events [Event List] through simple, step-by-step questions. Ensure your responses are concise, empathetic, and aligned with the user’s cognitive capacity; avoid complex sentence structures or ambiguous prompts.”

Adaptive control logic maps MMSE scores to interaction strategies. The MMSE threshold of 20 follows established clinical practice: scores of 20 to 26 indicate mild cognitive impairment, and scores below 20 indicate moderate-to-severe impairment per standard neuropsychological guidelines [20]. When scores fall within 20 to 26 (mild cognitive impairment), the system prioritizes open-ended guided prompts. When scores fall below 20 (moderate impairment), the system switches to closed-ended, yes or no, or multiple-choice prompts to reduce cognitive load. Generation parameters are dynamically adjusted across three dimensions: (1) prompt granularity, with step-by-step prompts triggered for scores of 20 or below; (2) response length, limited to 50 to 80 words for scores below 20 and 100 to 150 words for scores of 26 or above; and (3) memory retrieval cues, with more sensory and contextual cues incorporated for users with lower scores. In the present sample (MMSE range 11‐23), all participants fell within the below 20 or 20‐26 tiers; the 26-or-above response-length strategy was not triggered.

Recorded life events serve as foundational memory anchors. Initial acquisition follows a 2-step process: trained researchers conduct semistructured interviews of 30 to 45 minutes, and family members or caregivers complete a supplementary questionnaire. Storage uses a structured relational database organized hierarchically to support efficient retrieval. Cross-session updates follow 3 rules: supplemental update of newly mentioned events, semantic deduplication (cosine similarity threshold ≥0.8), and emotional tagging based on subsequent narratives. Feng et al [27] confirmed that emotional tagging enhances memory cue efficacy.

The system combines voice guidance with a visual timeline interface. The voice module delivers preloaded audio responses—recorded and stored locally on the device rather than generated via cloud text-to-speech. Emotional alignment is achieved through a rule-based mapping layer: the adaptive control logic classifies each interaction turn into 1 of 5 predefined emotional states (encouragement, gentle redirection, empathetic acknowledgment, memory affirmation, and session closure) based on MMSE score, topic category, and detected response latency, and selects the corresponding preloaded audio clip. The visual timeline interface presents life events in a hierarchical, chronologically organized format, reducing barriers for users with language and cognitive decline.

### Evaluation

#### Participants

Participants were recruited through online platforms (WeChat, Xiaohongshu, Bilibili, and Baidu) and in-person community outreach in Hebei, Henan, Tianjin, and Shandong provinces in China. Inclusion criteria: aged 65 to 80 years; clinically confirmed diagnosis of cognitive impairment by a neurologist or psychiatrist based on *Diagnostic and Statistical Manual of Mental Disorders, Fifth Edition* (DSM-5) criteria [28]; basic verbal communication abilities; and voluntary participation with written informed consent from participant and legal representative. Exclusion criteria: poor health requiring continuous medical care, severe psychiatric or behavioral symptoms, and severe uncorrectable sensory impairments.

Psychiatrists were recruited as independent evaluators. Inclusion criteria: valid clinical license in psychiatry, active practice in frontline psychiatric settings, at least 5 years of independent clinical experience, and clinical or research experience in geriatric psychiatry or neurocognitive disorders. Psychiatrists were excluded for conflicts of interest, personal relationships with participants, or inability to maintain blinding. All psychiatrists completed standardized rater training before formal scoring.

#### Ethical Considerations

This study received ethics approval from the institutional review board of Southwest University (SW20250115). All procedures complied with the Declaration of Helsinki. Each participant received about US $15 as compensation. The use of generative AI in memoir construction for individuals with reduced decision-making capacity raises ethical considerations beyond standard research ethics protocols. Four issues were addressed in the study design. First, informed consent explicitly covered AIGC: participants and their legal representatives were informed that the system may generate or paraphrase narrative text based on caregiver-verified memory anchors, and that this content would be incorporated into the memoir transcript. Second, output transparency was built into the caregiver verification workflow: caregiver-verified memory anchors were flagged in the system database, allowing a postsession distinction between content spoken directly by the participant, content paraphrased by the system, and content generated from verified anchors. Third, data governance: memoir-related inputs processed via the GPT-3.5 API were limited to anonymized life-event descriptors; no personally identifiable information was transmitted to the API, and all locally stored data are subject to deletion upon participant request. Fourth, caregiver verification was required before any memory anchor was added to the database, ensuring that AI-generated narrative content remained grounded in the participant’s actual experience rather than system-inferred assumptions [17,23].

#### Procedures

Researchers collected demographic and clinical information including age, sex, diagnosis, medical history, MMSE score, and years of education. Participants completed the Technology Use Attitudes and Proficiency Questionnaire and the Positive and Negative Affect Schedule (PANAS) and narrated a recent personal story for 5 minutes. Audio recordings were transcribed with consent and rated by psychiatrists using predefined criteria (see Multimedia Appendix 2 for MMSE).

Factor matching minimized baseline imbalance on 4 variables: MMSE total score, baseline memoir quality, years of education, and technology use attitudes and proficiency. Pairs were formed only after all 20 participants had completed baseline assessment. A researcher not otherwise involved in the subsequent group-assignment step reviewed the baseline profiles of all enrolled participants across these same 4 variables and manually grouped them into 10 pairs of participants judged to be most similar in overall baseline profile; this pairing was completed independently of, and prior to, any group assignment. Group allocation used a minimization procedure rather than simple randomization: for each of these 10 pairs, a researcher not otherwise involved in participant recruitment or assessment determined, in real time, which assignment of the 2 paired participants would minimize cumulative imbalance between the intervention and control groups across the 4 matching variables, and assigned the participants accordingly. This was a nonrandomized allocation procedure: assignment was determined by the minimization algorithm’s real-time balancing calculation rather than by chance (eg, a random-number generator or coin flip), and no random element was used at any stage of group assignment.

All participants completed a 10-minute oral memoir narration in the same controlled environment. The intervention group received assistance from the AIGC system, while the control group received an active control condition matched to the intervention group in session duration, number of prompting turns, frequency of audio feedback, device novelty, and researcher behavior, with only the AI-generated personalization and memory anchor–driven narrative removed. The entire process was audio-recorded and transcribed with participant consent. After the intervention, all participants completed the PANAS again. For the intervention group, AI-paraphrased content and AIGC were excluded from the transcript prior to rating, using the content-source flags recorded in the system database, so that only content spoken directly by the participant was retained for evaluation. Psychiatrists blinded to group assignment independently rated these participant-only transcripts; control-group transcripts were unaffected by this procedure, as they contained no AIGC. Intervention group participants also completed the System Usability Scale (SUS) and were interviewed about their experience (see Multimedia Appendix 3 for SUS).

All 10 intervention group participants completed a semistructured, one-on-one interview lasting approximately 15 to 20 minutes, using a guide (Multimedia Appendix 4) covering 5 domains: usability experience, emotional experience, perceived authenticity of AIGC, cognitive load, and willingness to continue use. Each domain began with a broad, nonleading question, followed by neutral, nondirective probes (eg, “could you say more about that?”) to elicit elaboration without suggesting a valenced response. Interviews were audio-recorded with consent. Because the interview sample coincided with the full intervention arm, no independent saturation criterion was applied.

#### Data Analysis

##### Quantitative Analysis

Affective measures were assessed using the PANAS [29], which contains two 10-item subscales measuring positive affect (PA) and negative affect (NA) on a 5-point scale (1=very slightly or not at all; 5=extremely). Both subscale scores range from 10 to 50.

We assessed the consistency of the physicians’ ratings, which serves as the foundation for the subsequent data analysis. Interrater reliability was good to excellent: composite memoir quality intraclass correlation coefficient (ICC)=0.82, narrative coherence ICC=0.83, richness of detail ICC=0.81, and personal uniqueness ICC=0.85.

Memoir quality was evaluated by psychiatrists across 3 dimensions: narrative coherence, richness of detail, and personal uniqueness, on a 5-point scale (1=very poor; 5=excellent). All psychiatrists rated transcripts in a fully blinded manner.

System usability was assessed using the simplified SUS [30], in which odd-numbered items are positively worded and even-numbered items are negatively worded; even-numbered items were reverse-scored prior to analysis, and the total score was computed as the sum of all adjusted item scores multiplied by 2.5, yielding a scale of 0 to 100. Technology use baseline was measured using a Technology Use Experience and Attitudes Questionnaire scored 0 to 40.

Analyses proceeded in four steps: (1) baseline comparability, (2) normality testing of within-group pre- and postdifference scores and of matched-pair between-group difference scores using the Shapiro-Wilk test, (3) within-group pre- and postcomparisons, and (4) between-group comparisons conducted on matched-pair differences, treating each matched pair as the unit of analysis. Because participants were allocated within matched pairs using a minimization procedure, the primary between-group analysis used paired *t* tests or Wilcoxon signed rank tests where normality of the matched-pair differences was violated. Parametric paired *t* tests were applied where normality held; Wilcoxon signed rank tests were applied elsewhere. All tests were 2-tailed (α=.05). Effect sizes are reported as Cohen *d* (normally distributed differences) or rank-biserial correlation *r* (nonnormal distributions).

To account for multiple comparisons across the 3 prespecified coprimary outcomes (PA, NA, and psychiatrist-rated memoir quality), we applied Benjamini-Hochberg correction (*Q*=0.05) to the 6 hypothesis tests involving the experimental condition (3 within-group and 3 between-group matched-pair comparisons). Baseline equivalence tests were excluded, as these test for nondifference and are not part of the confirmatory hypothesis family.

All quantitative analyses were conducted in R (version 4.3.2; R Foundation for Statistical Computing).

##### Qualitative Analysis

Audio recordings of the interviews were transcribed verbatim after participants gave consent, and researchers then checked the transcripts against the recordings and manually corrected obvious speech recognition errors and dialect expressions to ensure that the transcripts accurately reflected participants’ original words. Transcripts were deidentified before analysis, retaining only participant identification numbers and no personally identifiable information, and all coding was carried out in NVivo software (Lumivero) to facilitate code management and retrieval.

The analysis used reflexive thematic analysis. Coding was carried out independently by 2 researchers, each of whom read through all transcripts and assigned initial codes, without aiming in advance for agreement between their coding results. After independent coding was completed, the 2 researchers compared their coding results through discussion, examined each disagreement in turn, reinterpreted the material in light of its original context, and reached consensus through discussion or retained legitimate alternative interpretations, an approach consistent with the methodological position of reflexive thematic analysis, which treats coding as an interpretive and subjective process of knowledge production. Building on the coding, related codes were grouped and merged to progressively develop higher-level themes and subthemes, a thematic map was produced to show the hierarchy and relationships among themes, and a complete audit trail was maintained to document how the coding evolved from initial codes to final themes. Throughout the analysis, the emerging themes were continually checked against the original interview material to avoid overinterpretation detached from the data itself, and the recordings were revisited when necessary to verify tone and context.

## Results

### Overview

This study recruited 20 participants aged 67‐80 years. Baseline MMSE scores spanned the range of mild to moderate cognitive decline, and there was individual variation in baseline memoir quality (as assessed by a psychiatrist) as well as in attitudes toward and proficiency in technology use. Participants had 3‐12 years of education, reflecting the characteristics of an older population with diverse educational backgrounds. Detailed participant information is provided in Multimedia Appendix 5. There were no participant dropouts throughout the entire experimental process.

### Quantitative Results

Baseline comparability between the experimental (n=10) and control (n=10) groups was evaluated using paired *t* tests and standardized mean differences (SMDs). No statistically significant differences were found on any baseline variable (all *P*>.05), and all SMDs were negligible (|SMD|≤0.20), confirming successful group equivalence prior to the intervention (Table 1).

**Table 1.** Baseline demographic and clinical characteristics of experimental (n=10) and active control (n=10) participants in a single-session, nonrandomized, matched-pair evaluation of an AIGC^a^-based memoir-writing chatbot among older adults aged 67‐80 years with clinically diagnosed cognitive impairment, recruited from Hebei, Henan, Tianjin, and Shandong provinces, China.

Variable	Experimental, mean (SD)	Control, mean (SD)	SMD^b^	*t* test^c^ (*df*=9)	*P* value
MMSE^d^ total score	17.60 (3.84)	17.80 (3.79)	−0.052	−1.000	.34
Psychiatrist-rated baseline memoir quality	2.77 (0.63)	2.85 (0.54)	−0.128	−0.605	.56
Technology attitude and proficiency	18.10 (4.07)	18.20 (4.24)	−0.024	−0.287	.78
Education (years)	6.60 (2.76)	6.10 (2.23)	+0.199	1.861	.10
Age (years)	72.70 (3.74)	73.10 (3.90)	−0.105	−1.500	.17

a AIGC: AI-generated content.

b SMD: standardized mean difference.

c Paired *t* test.

d MMSE: Mini-Mental State Examination.

As shown in Table 2, normality of difference scores was assessed using the Shapiro-Wilk test, separately for within-group pre- and postdifferences and for the matched-pair between-group differences that form the basis of the primary analysis. Within-group differences were normally distributed only for PA in the experimental group (*W*=0.866; *P*=.09); all remaining within-group differences violated the normality assumption (*P*s<.05). Among the matched-pair between-group differences, PA (*W*=0.909; *P*=.28) was normally distributed and was analyzed with a paired *t* test; NA (*W*=0.843; *P*=.048) and psychiatrist-rated quality (*W*=0.833; *P*=.04) violated normality and were analyzed with the Wilcoxon signed rank test.

**Table 2.** Shapiro-Wilk tests of normality for within-group and matched-pair between-group difference scores in positive affect, negative affect, and psychiatrist-rated memoir quality, among 20 older adults with cognitive impairment participating in a single-session, nonrandomized, matched-pair evaluation of an AIGC^a^-based memoir-writing chatbot in China.

Difference tested	Group or level	*W*	*P* value	Normal (*P*≥.05)	Test selected
Positive affect	Within-group, experimental	0.866	.09	Yes	Paired *t* test
Positive affect	Within-group, control	0.774	.007	No	Wilcoxon
Negative affect	Within-group, experimental	0.768	.006	No	Wilcoxon
Negative affect	Within-group, control	0.731	.002	No	Wilcoxon
Psychiatrist quality	Within-group, experimental	0.802	.02	No	Wilcoxon
Psychiatrist quality	Within-group, control	0.794	.01	No	Wilcoxon
Positive affect	Between-group, matched-pair difference	0.909	.28	Yes	Paired *t* test
Negative affect	Between-group, matched-pair difference	0.843	.048	No	Wilcoxon
Psychiatrist quality	Between-group, matched-pair difference	0.833	.04	No	Wilcoxon

a AIGC: AI-generated content.

Within-group changes were examined separately for each group; all tests were 2-tailed (α=.05). In the experimental group, PA increased significantly from pretest (mean 23.50, SD 4.58) to posttest (mean 25.30, SD 4.74; Δ=1.80, 95% CI 0.55-3.05; *t*_9_=3.25; *P*=.01; Cohen *d*=1.03); the control group showed no significant change (Δ=−0.20; *W*=10.0; *P*=.54; *r*=−0.29). NA decreased significantly in the experimental group from pretest (mean 22.10, SD 5.22) to posttest (mean 18.40, SD 3.69; Δ=−3.70, 95% CI −7.19 to −0.21; *W*=3.0; *P*=.01; *r*=−0.89); the control group showed no significant change (Δ=0.50; *W*=0.0; *P*=.09; *r*=1.00). Psychiatrist-rated memoir quality, assessed from participant-generated content only, improved significantly in the experimental group from pretest (mean 2.775, SD 0.63) to posttest (mean 2.95, SD 0.73; Δ=0.175, 95% CI 0.054-0.296; *W*=0.0; *P*=.03; *r*=1.00); the control group showed no significant change (Δ=−0.05; *W*=2.5; *P*=.42; *r*=−0.50). Full within-group results are presented in Table 3. Additionally, Multimedia Appendix 6 presents separate ratings for AIGC.

**Table 3.** Within-group pre- to postintervention changes in positive affect, negative affect, and psychiatrist-rated memoir quality for the experimental (AIGC^a^ chatbot-assisted) and active control groups, in a one-time experimental evaluation among 20 older adults with cognitive impairment in China.

Outcome and group	Preintervention, mean (SD)	Postintervention, mean (SD)	Δ (95% CI)	Test statistic	*P* value	Effect size
Positive affect						
Experimental	23.50 (4.58)	25.30 (4.74)	1.80 (0.55 to 3.05)	*t*_9_=3.25	.01	*d*=1.03
Control	23.10 (4.25)	22.90 (3.96)	−0.20 (−1.14 to 0.74)	*W*=10.0	.54	*r*=−0.29
Negative affect						
Experimental	22.10 (5.22)	18.40 (3.69)	−3.70 (−7.19 to −0.21)	*W*=3.0	.01	*r*=−0.89
Control	25.30 (5.21)	25.80 (4.71)	0.50 (−0.01 to 1.01)	*W*=0.0	.09	*r*=1.00
Psychiatrist-rated quality						
Experimental	2.77 (0.63)	2.95 (0.73)	0.175 (0.054 to 0.296)	*W*=0.0	.03	*r*=1.00
Control	2.85 (0.54)	2.80 (0.57)	−0.05 (−0.16 to 0.06)	*W*=2.5	.42	*r*=−0.50

a AIGC: AI-generated content.

Between-group comparisons were conducted on matched-pair differences rather than independent-samples tests, because participants had been allocated within matched pairs, and each pair therefore constitutes the appropriate unit of comparison. Paired *t* tests were used for PA and psychiatrist-rated memoir quality, and the Wilcoxon signed rank test was used for NA, based on the normality of the matched-pair differences (Table 2). The experimental group showed significantly larger improvements than the control group across all 3 outcomes: PA (matched-pair Δ=2.00, 95% CI 0.31-3.69; *t*_9_=2.68; *P*=.03; Cohen *d*=0.85), NA (matched-pair Δ=−4.20, 95% CI −7.59 to −0.81; *W*=2.0; *P*=.008; rank-biserial *r*=−0.93), and psychiatrist-rated memoir quality, assessed from participant-generated content only (matched-pair Δ=0.225, 95% CI 0.093-0.357; *W*=0; *P*=.02; rank-biserial *r*=1.00; Table 4).

**Table 4.** Matched-pair between-group comparison of change scores in positive affect, negative affect, and psychiatrist-rated memoir quality between the AIGC^a^ chatbot-assisted experimental group and the active control group, among 10 matched pairs of older adults with cognitive impairment in a one-time experimental evaluation in China.

Outcome	Matched-pair Δ, mean (SD)	95% CI	Test statistic	*P* value	Effect size
Positive affect	2.00 (2.36)	0.31 to 3.69	*t*_9_=2.68	.03	*d*=0.85
Negative affect	−4.20 (4.73)	−7.59 to −0.81	*W*=2.0	.008	*r*=−0.93
Psychiatrist-rated quality	0.225 (0.184)	0.093 to 0.357	*W*=0	.02	*r*=1.00

a AIGC: AI-generated content.

To account for multiple comparisons across the 3 prespecified coprimary outcomes (PA, NA, and psychiatrist-rated memoir quality), we applied Benjamini-Hochberg correction (*Q*=0.05) to the 6 hypothesis tests involving the experimental condition (3 within-group and 3 between-group matched-pair comparisons). Baseline equivalence tests in Table 1 were excluded, as these test for nondifference and are not part of the confirmatory hypothesis family. All 6 tests remained significant after correction (*q*≤0.031; Table 5). The 3 within-group control-condition comparisons, reported descriptively, remained nonsignificant (*P*≥.089).

**Table 5.** Benjamini-Hochberg (BH) false discovery rate correction applied to the 6 confirmatory hypothesis tests (3 within-group and 3 matched-pair between-group comparisons) for positive affect, negative affect, and psychiatrist-rated memoir quality, among 20 older adults with cognitive impairment participating in a single-session, nonrandomized, matched-pair evaluation of an AIGC^a^-based memoir-writing chatbot in China.

Test	Raw *P* value	Rank	BH-adjusted *q*	Significant (*q*<0.05)
Between-group, negative affect	.008	1	.024	Yes
Within-group, positive affect	.01	2	.024	Yes
Within-group, negative affect	.01	3	.024	Yes
Between-group, psychiatrist quality	.02	4	.024	Yes
Between-group, positive affect	.03	5	.030	Yes
Within-group, psychiatrist quality	.03	6	.031	Yes

a AIGC: AI-generated content.

System usability was assessed using the simplified SUS [30], in which odd-numbered items are positively worded and even-numbered items are negatively worded; even-numbered items were reverse-scored prior to analysis. The mean SUS total score among intervention group participants was 92.2 (SD 5.1; range 82.5‐97.5), which exceeds the widely used acceptability threshold of 68 and falls within the “excellent” range (>85) for dementia-assistive technologies [31,32]. Individual scores ranged from 82.5 (Participant 10) to 97.5 (Participant 6 and Participant 7), with no participant scoring below the acceptable threshold. Subscale analysis following Orgeta et al [22] yielded a mean effectiveness score (items 1, 3, 5, 7, and 9) of 4.80 (SD 0.19) and a mean simplicity score (items 2, 6, 8, and 10) of 4.47 (SD 0.40), both on a 1‐5 scale, indicating consistently high perceived effectiveness and adequate simplicity across participants.

The experimental group showed greater improvement than the active control group across PA, NA, and psychiatrist-rated memoir quality. Because the control condition was matched to the intervention condition in session duration, number of prompting turns, frequency of audio feedback, device exposure, and researcher behavior, the observed differences are less likely to be explained solely by interaction time or device novelty. However, the fidelity of the manually delivered control condition was not independently assessed, and the study was not designed to determine which specific component of the AIGC intervention—personalized prompting, memory-anchor retrieval, narrative generation, or their combination—accounted for the observed differences. The findings should therefore be interpreted as preliminary evidence concerning the integrated intervention rather than as proof of the effectiveness of any single system component.

Subgroup patterns offer additional insight. In the present sample, 12 of 20 participants (experimental: n=6; control: n=6) had MMSE scores below 20 (range 11‐18), triggering the closed-ended, high-proactivity interaction mode; the remaining 8 (experimental: n=4; control: n=4) had MMSE scores of 20‐23, triggering the open-ended, keyword-based mode. Participants in the lower MMSE tier appeared to benefit more from AI-driven narrative generation, while those in the higher tier responded better to keyword-based prompting. This is consistent with cognitive load theory [33]: when residual capacity is limited, proactive narrative scaffolding reduces the compositional burden and allows users to engage with their memories without being overwhelmed. Users with greater preserved capacity may find fully automated narrative generation less engaging, though the mechanism remains speculative. These patterns tentatively suggest that cognitive severity may shape the appropriate level of AI proactivity, and that a uniform interaction mode would be inadequate for this population. Given the small *n* within each tier (6 and 4 per group), these observations are descriptive and should not be interpreted as subgroup-level statistical conclusions.

### Qualitative Results

First, most participants reported some initial trial and error during the first 1 or 2 conversational turns within the session, but that they became more familiar with the system as the number of turns increased. (Throughout this section, references to “uses” or “times” refer to individual conversational turns or exchanges within the single, approximately 10-minute session, not separate sessions on different occasions.) This pattern of initial difficulty followed by increasing fluency recurred throughout the interviews, particularly with regard to the timing and triggering of voice commands. Many participants mentioned that during their first few turns they were unsure “when they should speak and when they should wait for the system to finish speaking,” and this uncertainty clearly diminished as the session progressed.

*At the beginning I did not really know how to use it, but fortunately the system was simple, and after using it a few times I got the hang of it . Later on I did not really get stuck anymore*. (*The references to “few times” by the participants here refer to multiple rounds of dialogue, rather than the conduct of multiple experiments*.)

[Participant 2]

Second, in terms of emotional experience, participants’ descriptions showed a fairly consistent positive tendency. Most described the act of “telling their story” itself as a process of being taken seriously and being listened to. Some participants compared this with their everyday communication with family and friends, feeling that the system “does not interrupt” and “does not get impatient,” which they saw as setting it apart from interpersonal communication.

It was a lot of fun. I think it gave me a fairly good window that made it convenient for me to express myself. How should I put it, it lets me talk, and I mean that in a meaningful sense, like confiding in someone, it made that more convenient. It was very patient, it did not interrupt me, and it let me finish speaking slowly.

[Participant 1]

Next is the issue of autonomy. This was the theme discussed in the greatest depth in the interviews, and the one on which participants’ attitudes diverged most clearly. Most participants felt that even though the system organized and rewrote their spoken content, the resulting story still “belonged to them,” because the core facts and emotions came from themselves and the system only played a supporting role in “polishing” or “recording” it.

I think the story is my own. As for the final generated result, when I look at most of it, I feel it matches what I wanted to express, and there is not really a problem.

[Participant 3]

However, a small number of participants (such as Participant 6 and Participant 8) expressed more nuanced reservations, noting that the wording the system produced after rewriting sometimes differed from their usual way of expressing themselves. They hoped to have the opportunity to check or revise the rewritten portions rather than simply accepting the final text passively.

Even though on the whole I feel most of it is fitting, to be honest some of the details still feel a bit off to me. For example that table, the one it generated, is not entirely how I wanted it to look. But it is true that if you wanted it to be completely accurate, that would probably take a lot of effort.

[Participant 6]

Regarding the system’s design of adjusting its prompting style according to cognitive state (open-ended prompts vs stepwise closed-ended prompts), participant feedback showed a divergent trend related to their self-reported cognitive burden. Among participants with lower cognitive burden (corresponding to the MMSE stratum of 20 to 23), some felt that stepwise prompting was “a bit wordy” and preferred to speak directly and freely, feeling that their train of thought was interrupted.

I feel it could be more concise. Sometimes it says things that feel unnecessary, and I do not think those are really needed.

[Participant 7]

Participants with higher cognitive burden (corresponding to an MMSE score below 20), by contrast, were more inclined to report that stepwise prompting “was a huge help,” reducing the pressure of organizing their language and easing the anxiety of “not knowing where to start.”

I think it was extremely useful, it helped me a lot. Otherwise, having to do everything myself would really have been difficult.

[Participant 4]

This divergence corroborates the original intent behind this study’s MMSE stratified design, namely, that greater cognitive burden calls for more structured guidance. It also suggests that future versions could allow users to actively switch the density of prompting during use rather than having it entirely preset by the system, so as to accommodate the desire for autonomous expression among participants with milder impairment. It should be noted that because this study is only a preliminary formative study with a small sample size, it is difficult to support adequate subgroup analysis, and the description above should be treated only as an indication of a trend.

With regard to willingness to continue using the system, all participants expressed a positive attitude, but this positive attitude often came with specific conditions. For example, whether the system could remember what had previously been said (to avoid the fatigue of repeating the same content) and whether interest would wane once the novelty faded.

It is easy to use, a lot of fun, and interesting, but I am not sure whether it can keep a record. I hope it can keep recording continuously and save all of it permanently.

[Participant 10]

One participant, Participant 8, linked the willingness to continue using the system to a deeper motivation, that of “leaving something behind for later generations,” which goes beyond a purely instrumental evaluation of the tool and positions the system as a medium for recording and passing things on rather than simply a companion or entertainment tool.

After I am gone, this will count as a kind of digital photo album too, and that is a good thing.

[Participant 8]

## Discussion

### Principal Findings

Improvements in PA and NA, as measured by PANAS, suggest that the system may have supported emotional well-being during the memoir-writing session. This aligns with the KE component of the design framework, which translated affective needs (eg, the desire for recognition and anxiety about forgetting) into features such as empathetic audio feedback and sensory memory cues. Pappadà et al [7] noted that dementia-assistive technologies tend to prioritize cognitive function while underserving emotional needs; these results suggest that emotional engineering principles may contribute meaningful affective benefits.

The empirical findings, together with the design process, suggest 3 implications for future dementia-assistive AIGC systems. First, AI proactivity should adapt to cognitive capacity: more severe impairment calls for greater AI narrative initiative, while milder impairment is better served by user-driven prompting that preserves authorial agency, or the user’s sense that the memoir remains their own story. Second, memoir tools should support affective safety alongside memory retrieval. Third, long-term personalization requires a structured life-event memory system with caregiver verification mechanisms and ethical safeguards to ensure that AIGC remains grounded in the user’s actual experience. The multimethod design framework (KE, QFD, AD, and TOPSIS) remains relatively underexplored in dementia-assistive technology [21] and may offer a reproducible starting point for similar work.

### Comparison to Prior Work

This study extends prior work on AI-mediated reminiscence and narrative support for older adults [5,12,27]. More broadly, multimodal AI-driven platforms have also shown promise in supporting expressive and creative interventions for home-based older adult care, such as interactive art therapy systems [34], reinforcing the potential of AI-based creative engagement to benefit older populations beyond memoir writing specifically. Wang et al [12] demonstrated that AI-driven multimodal photo albums support personalized reminiscence in cognitively intact older adults; our study extends this to individuals with diagnosed cognitive impairment, using MMSE scores to drive adaptive interaction control. Feng et al [27] found that contextual and multimodal stimuli enhanced engagement in people with dementia during human-robot interaction; our results suggest that similar multimodal principles may be effective in chatbot-mediated memoir writing. Chang et al [35] further demonstrated that reinforcement learning-based AI can deliver personalized adaptive support in dementia care contexts, reinforcing the value of dynamic AI adaptation for this population.

The present findings also converge with recent longitudinal evidence. Kang et al (2025) [36] found that regular AI care calls over 7 months significantly reduced depression scores and improved memory function in 80 community-dwelling individuals with dementia. That study found that sex and education level moderated intervention outcomes, paralleling our subgroup finding that cognitive severity shapes response to AI interaction and reinforcing the view that individual characteristics meaningfully affect how people with dementia benefit from AI-based support.

The findings also speak to HCI discussions of asymmetric human-AI collaboration [10,37], in which AI systems dynamically compensate for differential user capabilities. Here, narrative initiative is inversely calibrated to residual cognitive capacity, so the AI assumes greater responsibility where the user needs it most. This contrasts with many prior dementia-assistive systems that rely on relatively fixed interaction modes [7]. Whether similar mechanisms generalize to other cognitive assessment instruments remains a question for future work.

### Limitations

However, this study also has some limitations. First, the sample size (n=20; 10 per arm) is small. Effect sizes derived from small samples are inherently unstable, and the Benjamini-Hochberg correction applied to the 6 confirmatory hypothesis tests should be read with caution: because within- and between-group tests were derived from the same 10 participants per arm rather than independent samples, the positive regression–dependence assumption underlying the procedure was plausible but not formally verified, and several outcomes assessed with Wilcoxon signed rank tests at n=9‐10 yield *P* and *q* values that take on a limited set of discrete values. The correction should be read as a conservative response to multiplicity concerns rather than a substitute for replication in a larger, adequately powered, multisite trial.

Second, this study used a nonrandomized design: group allocation used a minimization procedure rather than simple or block randomization. This method was selected specifically to ensure equivalence on 4 baseline variables, given the small overall sample size, and is a recognized alternative to simple randomization in small trials. However, it differs from simple randomization in that assignment for later-entered pairs is partly determined by the accumulated composition of previously assigned groups rather than by chance alone. As a result, unmeasured or unbalanced covariates could, in principle, be associated with allocation in ways that pure randomization is designed to prevent, and a person with knowledge of the algorithm and running group totals could in principle anticipate upcoming assignments. Larger-scale replications should use simple or stratified block randomization with formal allocation concealment to strengthen causal inference.

Third, because participants were allocated within matched pairs, all confirmatory hypothesis tests were analyzed using paired methods (paired *t* tests or Wilcoxon signed rank tests on matched-pair differences) rather than independent-samples tests, consistent with the matched design. This is a more conservative and design-appropriate approach than treating change scores as independent observations; all 6 confirmatory comparisons remained statistically significant under this analysis and after Benjamini-Hochberg correction, with moderate-to-large effect sizes (Cohen *d*=0.85‐1.03; rank-biserial *r*=−0.93 to 1.00).

Fourth, this was a one-time experimental evaluation without follow-up assessment. This has 2 consequences. It precludes conclusions about the durability of affective and narrative gains; whether benefits persist, accumulate with repeated use, or diminish with habituation is unknown. It also means the high SUS scores (mean 92.2, SD 5.1) should be interpreted cautiously: single exposure to a novel system can temporarily inflate satisfaction ratings, and older adults with cognitive impairment are additionally prone to acquiescence bias, meaning the tendency to agree with survey items regardless of content, particularly under limited cognitive resources [38]. Holden [30] noted similar interpretive challenges applying the SUS in this population. Longitudinal, multisession usability assessment is needed to obtain a more reliable estimate of system acceptability.

Fifth, participants were recruited exclusively from 4 Chinese provinces, with a mean education level of approximately 6 years and corresponding technology-use attitudes. System performance may differ in populations with higher digital literacy, different cultural or linguistic backgrounds, or distinct dementia etiologies, and these findings should not be generalized beyond this context without further testing.

Sixth, the system’s core AIGC engine relies on GPT-3.5. GPT-3.5 represented a relatively advanced, readily available commercial large language model at the time this system was developed, and its selection was reasonable within that context. However, given the pace at which generative AI models have since evolved, findings obtained with this specific model may not generalize to systems built on newer architectures. Replication with current-generation models is needed, alongside systematic safety evaluation under diverse real-world conditions, which has not yet been conducted for either version.

Seventh, the control condition requires clarification and cautious interpretation. As described in the Procedures section, the control group received an active control matched to the intervention group on session duration, number of prompting turns, frequency of audio feedback, device novelty, and researcher behavior, with only the AI-generated personalization and memory anchor–driven narrative content removed. This design was intended to isolate the specific contribution of AI-generated scaffolding rather than general attention or novelty effects. However, no formal fidelity check (eg, independent rating of researcher adherence to the matched protocol) was conducted to confirm that the manually delivered control condition in fact replicated the intervention’s interaction structure and pacing. Residual, unmeasured differences in perceived responsiveness or engagement between human-delivered and AI-delivered support therefore cannot be fully excluded, and this should be addressed in future trials through structured fidelity monitoring or a non-AI digital comparator.

Eighth, psychiatrist-rated memoir quality was assessed from transcripts with AI-paraphrased content and AIGC excluded, so that scores reflect participant-generated narrative content specifically rather than the system’s language capabilities.

### Conclusions

AI-assisted memoir construction for people with reduced decision-making capacity raises important questions about authorship, narrative authenticity, and data governance. As described in the Ethical Considerations section, this study addressed these concerns through explicit consent for AIGC, caregiver verification of generated narratives, anonymized API data transmission, and postsession transparency about system outputs. Future deployments should extend these safeguards to longitudinal use by formalizing policies for data retention, access, revision, and deletion [17,23].

Future studies should evaluate the system across multiple sessions and with stronger comparison conditions, such as structured human prompting or non-AI digital memoir tools, to better isolate the contribution of AI-generated scaffolding. A larger and more diverse randomized controlled trial would also enable subgroup analyses by dementia etiology, severity, education, and cultural context. Finally, because the current MMSE-based adaptation logic remains relatively coarse, future versions could incorporate real-time behavioral signals, such as response latency, lexical complexity, and speech-detected emotional tone, to support more fine-grained within-session adaptation [39]. Together, these steps would help move the system from a promising single-session prototype toward a clinically useful and ethically accountable long-term reminiscence support tool.

## Supplementary material

10.2196/83428 Multimedia Appendix 1 Hardware prototype.

10.2196/83428 Multimedia Appendix 2 Items of the Technology Use Experience and Attitudes Questionnaire.

10.2196/83428 Multimedia Appendix 3 Items of the Simplified System Usability Scale.

10.2196/83428 Multimedia Appendix 4 Semistructured interview guide.

10.2196/83428 Multimedia Appendix 5 Baseline demographic and clinical characteristics of 20 individually matched participants.

10.2196/83428 Multimedia Appendix 6 Sensitivity comparison of psychiatrist-rated memoir quality scored from human-only versus AI-inclusive transcript content among 10 experimental-group participants.

## Abbreviations

AD: Axiomatic Design
AIGC: AI-generated content
DSM-5: Diagnostic and Statistical Manual of Mental Disorders, Fifth Edition
HCI: human-computer interaction
ICC: intraclass correlation coefficient
KE: Kansei Engineering
MMSE: Mini-Mental State Examination
NA: negative affect
PA: positive affect
PANAS: Positive and Negative Affect Schedule
QFD: Quality Function Deployment
SMD: standardized mean difference
SUS: System Usability Scale
TOPSIS: Technique for Order of Preference by Similarity to Ideal Solution
