# Supplementary material for: Personalized Intelligent Chatbot Based on AI-Generated Content Assists Memoir Writing for Older Adults With Cognitive Impairment: Mixed Methods Study
Source: JMIR Hum Factors. 2026 Aug 25;13:e83428. doi: 10.2196/83428 (PMC13505345; doi:10.2196/83428)
Supplement: Supplementary file 1 [file humanfactors-v13-e83428-s001.docx]

As shown in Figures S1 and S2, the hardware prototype uses the STC15L2K16S2 microcontroller as its central processing unit, an ESP-01SF WiFi module for wireless communication, and a GD5600 MP3 decoder chip for local audio playback. The STC15L2K16S2 is an 8-bit, 8051-architecture microcontroller with 16 KB of Flash memory and 2 KB of SRAM, an operating voltage range of 2.2 to 3.6 V, an integrated oscillator and reset logic, and two UART serial interfaces that allow simultaneous communication with the WiFi and voice modules; it parses external commands, manages serial communication, and issues control signals to the voice module and emotional-feedback circuitry. The ESP-01SF integrates the ESP8266 SoC, a 32-bit microcontroller with a WiFi transceiver and full TCP/IP support, communicating with the main controller via UART ; in this design the ESP8266 is configured as a server and the companion smartphone application as the client, enabling real-time control and data exchange. The GD5600 MP3 decoder supports playback of MP3 and WAV files stored in internal flash memory or on a TF card, interfaces with the main controller via UART, and can be commanded to play specific pre-loaded audio tracks corresponding to particular verbal responses or emotional states; playback is triggered through simple serial commands, with logical input combinations (a high/low input triggers a play command and the corresponding voice track, while no input produces no audio output) governing which track is played. Each module was soldered and bench-tested independently — the microcontroller, WiFi, and voice modules were verified using AT commands, and the GD5600 voice module was tested with serial commands to confirm correct audio triggering — before the components were assembled onto the robot chassis and interconnected; the companion smartphone application initiated interaction over WiFi, with the robot responding to voice commands and simulating emotional responses through audio and LED indicators. This early hardware prototype did not include locomotion capabilities. All modules were selected for low power consumption and compatibility with the sensory and motor limitations common among elderly users [31].

***Figure S1. AI-generated concept rendering of the home-use assistive companion device designed to deliver an AIGC-based memoir-writing chatbot intervention for older adults with cognitive impairment, illustrating its intended in-home deployment context.***

***Figure S2. Hardware components of the companion device used to deliver the AIGC-based memoir-writing chatbot intervention to older adults with cognitive impairment in this single-session, matched-pair evaluation: (1) the ESP-01SF WiFi module integrated in the system; (2) the voice module schematic based on the GD5600 chip.***
