# Supplementary material for: Personalized Intelligent Chatbot Based on AI-Generated Content Assists Memoir Writing for Older Adults With Cognitive Impairment: Mixed Methods Study
Source: JMIR Hum Factors. 2026 Aug 25;13:e83428. doi: 10.2196/83428 (PMC13505345; doi:10.2196/83428)
Supplement: Supplementary file 2 [file humanfactors-v13-e83428-s002.docx]

***Items of the Technology Use Experience and Attitudes Questionnaire administered at baseline to all 20 older adult participants (experimental and active control groups) with cognitive impairment prior to a single-session memoir-writing chatbot intervention in China.***

| **Item** | **Question** | **Response Options (Score)** |
| --- | --- | --- |
| **Section I: Usage Frequency** | | |
| 1 | How often do you use a mobile phone (including senior phones) to make or receive calls? | ☐ Multiple times per day (4)  ☐ Once per day (3)  ☐ Several times per week (2)  ☐ A few times per month or less (1)  ☐ Never (0) |
| 2 | How often do you watch videos on a phone or tablet (e.g., on Douyin, WeChat)? | ☐ Multiple times per day (4)  ☐ Once per day (3)  ☐ Several times per week (2)  ☐ A few times per month or less (1)  ☐ Never (0) |
| 3 | How often do you browse news, read articles, or search for information using a phone or computer? | ☐ Multiple times per day (4)  ☐ Once per day (3)  ☐ Several times per week (2)  ☐ A few times per month or less (1)  ☐ Never (0) |
| **Section II: Ability and Confidence** | | |
| 4 | Adjust the phone’s volume or brightness. | ☐ No difficulty (4)  ☐ Very slight difficulty (3)  ☐ Some difficulty but can complete (2)  ☐ Very difficult, need help (1)  ☐ Never done this (0) |
| 5 | Connect to home Wi-Fi. | ☐ No difficulty (4)  ☐ Very slight difficulty (3)  ☐ Some difficulty but can complete (2)  ☐ Very difficult, need help (1)  ☐ Never done this (0) |
| 6 | Send a voice message on WeChat. | ☐ No difficulty (4)  ☐ Very slight difficulty (3)  ☐ Some difficulty but can complete (2)  ☐ Very difficult, need help (1)  ☐ Never done this (0) |
| 7 | Install a new app on the phone. | ☐ No difficulty (4)  ☐ Very slight difficulty (3)  ☐ Some difficulty but can complete (2)  ☐ Very difficult, need help (1)  ☐ Never done this (0) |
| **Section III: Emotional Attitudes** | | |
| 8 | I think learning to use new electronic devices is fun. | ☐ Strongly agree (4)  ☐ Agree (3)  ☐ Neutral/unsure (2)  ☐ Disagree (1)  ☐ Strongly disagree (0) |
| 9 | I feel nervous or overwhelmed when electronic devices malfunction. | ☐ Strongly agree (0)  ☐ Agree (1)  ☐ Neutral/unsure (2)  ☐ Disagree (3)  ☐ Strongly disagree (4) |
| 10 | Overall, I think technology makes my life more convenient. | ☐ Strongly agree (4)  ☐ Agree (3)  ☐ Neutral/unsure (2)  ☐ Disagree (1)  ☐ Strongly disagree (0) |
