# Supplementary material for: Personalized Intelligent Chatbot Based on AI-Generated Content Assists Memoir Writing for Older Adults With Cognitive Impairment: Mixed Methods Study
Source: JMIR Hum Factors. 2026 Aug 25;13:e83428. doi: 10.2196/83428 (PMC13505345; doi:10.2196/83428)
Supplement: Supplementary file 3 [file humanfactors-v13-e83428-s003.docx]

***Items of the Simplified System Usability Scale (SUS) administered to 10 experimental-group participants (older adults with cognitive impairment) immediately following a single-session, AIGC chatbot-assisted memoir-writing intervention in China***

| **Item** | **Statement** | **Rating (1–5)** | **Scored Value** |
| --- | --- | --- | --- |
| 1 | I think that I would like to use this system frequently. |  |  |
| 2 | I found the system unnecessarily complex. |  |  |
| 3 | I thought the system was easy to use. |  |  |
| 4 | I think that I need technical support to use this system. |  |  |
| 5 | I found the various functions in this system were well integrated. |  |  |
| 6 | I thought there was too much inconsistency in this system. |  |  |
| 7 | I imagine most people would learn to use this system very quickly. |  |  |
| 8 | I found the system very cumbersome to use. |  |  |
| 9 | I felt very confident using the system. |  |  |
| 10 | I needed to learn a lot of things before I could get going with this system. |  |  |
| **Total** |  |  |  |
