# Supplementary material for: Personalized Intelligent Chatbot Based on AI-Generated Content Assists Memoir Writing for Older Adults With Cognitive Impairment: Mixed Methods Study
Source: JMIR Hum Factors. 2026 Aug 25;13:e83428. doi: 10.2196/83428 (PMC13505345; doi:10.2196/83428)
Supplement: Supplementary file 4 [file humanfactors-v13-e83428-s004.docx]

## ***Semi-structured interview guide administered to 10 experimental-group participants (older adults with cognitive impairment) after completion of the System Usability Scale, following a single-session, AIGC chatbot-assisted memoir-writing intervention in China; interviews lasted approximately 15–20 minutes and covered five domains: usability, emotional experience, perceived authenticity, cognitive load, and willingness to continue use***

## **Opening (rapport-building; non-evaluative)**

*"Thank you for completing the questionnaire. Now I'd like to talk with you about your experience using the system just now, and sharing your stories. There are no right or wrong answers — please share as much or as little as you'd like."*

**Q0. "Looking back on this session, how would you describe your overall experience?"**

*(Open-ended starting point; does not presuppose a positive or negative valence. Subsequent probes follow whatever the participant raises.)*

*Probe: "You mentioned [reflect participant's own keyword] — could you tell me more about that?"*

*Probe: "Was there anything else you noticed, besides that?"*

## **Theme 1: Usability Experience**

**Q1. "While you were using the system, was there a part that felt easy to use, or a part that felt awkward or difficult?"**

*Probe: "Could you tell me more about what that was like?"*

*Probe: "How did that get resolved, or did you just move past it?"*

**Q2. "Was there ever a time the system didn't understand what you meant, or responded with something that wasn't quite what you wanted to say? What happened, and how did you handle it?"**

*Probe: "Did that happen a few times, or just once or twice?"*

*Probe: "How did you feel in that moment?"*

**Q3. "Compared with things like using your phone or watching TV, did using this system feel about the same, or different? In what way?"**

*Probe: "Could you give me a specific example?"*

## **Theme 2: Emotional Experience**

**Q4. "What was it like to talk with the system and share stories about your past?"**

*Probe: "When did you start feeling that way?"*

*Probe: "Could you say more about that?"*

**Q5. "How is that similar to, or different from, talking with family or friends about things from the past?"**

*Probe: "Could you give an example to compare the two?"*

**Q6. "When the system responded to you — for example, with comforting or encouraging words — what did that feel like?"**

*(Probe with "could you say more about that?" rather than a directional probe such as "didn't that feel warm?")*

*Probe: "Could you give an example that stood out to you?"*

*Probe: "How did you respond in that moment?"*

**Q7. "Was there a moment during the whole process that stood out to you — whether it felt comfortable or uncomfortable?"**

*Probe: "What exactly happened at that point?"*

*Probe: "Looking back on it now, has how you feel about it changed at all?"*

## **Theme 3: Authenticity and Trust**

**Q8. "After the system organized and wrote down what you shared, did it still feel like your own story?"**

*Probe: "What gave you that impression?"*

*Probe: "Could you point to a specific part and tell me more?"*

**Q9. "Did what the system wrote match what you originally meant to say, or were there parts that were a bit different? Could you give an example?"**

*Probe: "For the parts that were different, what did you do — did you point it out and have it changed?"*

**Q10. "Did it matter to you which parts were things you said yourself, versus parts the system added or rephrased?"**

*Probe: "Could you say more about why it did or didn't matter to you?"*

## **Theme 4: Cognitive Load**

**Q11. "Were the questions the system asked easy to understand? Was there ever a time a question felt too roundabout or hard to follow?"**

*Probe: "Could you give a specific question as an example?"*

*Probe: "How did you handle that — did you ask it to repeat, or just skip past it?"*

**Q12. "The system asks questions one step at a time — did you find that helpful, or did it feel repetitive and make you want to just talk freely?"**

*Probe: "Could you say more about which aspect you mean?"*

## **Theme 5: Continued Use and Suggestions for Improvement**

**Q13. "If you could keep using this system going forward, would you want to? Why or why not?"**

*Probe: "Under what circumstances would you be more likely to want to use it?"*

**Q14. "If the system could be improved, what would you like to see added or changed?"**

*Probe: "Could you tell me more about where that idea came from?"*

**Q15. "Is there anything else on your mind that I haven't asked about, but that you'd like to share?"**

*Probe: "(If participant responds) Could you say more about that?"*
