# Supplementary material for: Personalized Intelligent Chatbot Based on AI-Generated Content Assists Memoir Writing for Older Adults With Cognitive Impairment: Mixed Methods Study
Source: JMIR Hum Factors. 2026 Aug 25;13:e83428. doi: 10.2196/83428 (PMC13505345; doi:10.2196/83428)
Supplement: Supplementary file 5 [file humanfactors-v13-e83428-s005.docx]

***Baseline demographic and clinical characteristics (MMSE score, psychiatrist-rated baseline memoir quality, technology attitude and proficiency, years of education, and age) of 20 individually matched participants (P1–P20; 10 experimental, 10 active control) with clinically diagnosed cognitive impairment, enrolled in a*** one-time experimental evaluation  ***in Hebei, Henan, Tianjin, and Shandong provinces, China.***

| **Participant** | **MMSE Score** | **Baseline Memoir Quality (Psychiatrist-Rated)** | **Technology Attitude and Proficiency** | **Years of Education** | **Age** |
| --- | --- | --- | --- | --- | --- |
| P1 | 21 | 3.50 | 22 | 12 | 79 |
| P2 | 17 | 3.00 | 20 | 6 | 73 |
| P3 | 20 | 3.00 | 23 | 6 | 77 |
| P4 | 15 | 2.50 | 16 | 9 | 75 |
| P5 | 16 | 2.50 | 13 | 9 | 73 |
| P6 | 17 | 2.50 | 14 | 6 | 73 |
| P7 | 23 | 3.50 | 23 | 6 | 72 |
| P8 | 22 | 3.50 | 21 | 3 | 70 |
| P9 | 11 | 1.75 | 15 | 3 | 67 |
| P10 | 14 | 2.00 | 14 | 6 | 68 |
| P11 | 22 | 3.00 | 22 | 10 | 80 |
| P12 | 16 | 3.00 | 19 | 5 | 74 |
| P13 | 20 | 3.50 | 23 | 6 | 76 |
| P14 | 15 | 2.50 | 16 | 9 | 75 |
| P15 | 16 | 2.25 | 15 | 7 | 75 |
| P16 | 18 | 3.00 | 14 | 6 | 73 |
| P17 | 23 | 3.00 | 23 | 6 | 73 |
| P18 | 22 | 3.75 | 23 | 3 | 70 |
| P19 | 12 | 2.00 | 14 | 3 | 67 |
| P20 | 14 | 2.50 | 13 | 6 | 68 |
