# Supplementary material for: Personalized Intelligent Chatbot Based on AI-Generated Content Assists Memoir Writing for Older Adults With Cognitive Impairment: Mixed Methods Study
Source: JMIR Hum Factors. 2026 Aug 25;13:e83428. doi: 10.2196/83428 (PMC13505345; doi:10.2196/83428)
Supplement: Supplementary file 6 [file humanfactors-v13-e83428-s006.docx]

**Sensitivity comparison of psychiatrist-rated memoir quality scored from human-only versus AI-inclusive transcript content among 10 experimental-group participants**

| **Measure** | **Value** |
| --- | --- |
| Mean (SD), human-only content | 2.95 (0.65) |
| Mean (SD), AI-inclusive content | 3.48 (0.55) |
| Mean paired difference (SD) | 0.525 (0.275) |
| 95% CI | [0.33, 0.72] |
| Paired t-test | t(9) = 6.03, P < .001 |
| Wilcoxon signed-rank test | W = 0.0, P = .002 |
| Effect size | Cohen's d = 1.91; 10/10 participants scored higher on AI-inclusive content |

We compared scores derived from human-only transcripts (AI-generated and AI-paraphrased content removed) against scores derived from AI-inclusive transcripts (the same transcripts prior to content-source exclusion), for the same 10 experimental-group participants, rated by the same blinded psychiatrists using identical criteria. Mean ratings were higher for AI-inclusive content (3.48, SD 0.55) than for human-only content (2.95, SD 0.65), with a mean paired difference of 0.53 points (SD 0.28, 95% CI 0.33–0.72). This difference was consistent in direction across all 10 participants, with no exceptions. A paired t-test (t(9) = 6.03, P < .001) and a Wilcoxon signed-rank test (W = 0.0, P = .002) both indicated a difference between the two scoring conditions, and the effect size was large (Cohen's d = 1.91). This comparison is presented descriptively to characterize the magnitude and consistency of the difference between the two content sources; it is not intended as a formal hypothesis test of the primary study outcomes, which are reported separately using human-only content throughout the manuscript.
